# Assembly of Conducting Polymer and Biohydrogel for the Release and Real-Time Monitoring of Vitamin K3

**DOI:** 10.3390/gels4040086

**Published:** 2018-11-28

**Authors:** Brenda G. Molina, Eva Domínguez, Elaine Armelin, Carlos Alemán

**Affiliations:** 1 Departament d’Enginyeria Química, EEBE, Universitat Politècnica de Catalunya, C/Eduard Maristany, 10-14, Ed. I2, 08019, Barcelona, Spain; eva.domher@hotmail.com (E.D.); elaine.armelin@upc.edu (E.A.); 2 Barcelona Research Center for Multiscale Science and Engineering, Universitat Politècnica de Catalunya, C/Eduard Maristany, 10-14, Ed. C, 08019, Barcelona, Spain

**Keywords:** conducting polymer, electroactive hydrogel, flexible electrode, menadione, poly(3,4-ethylenedioxythiophene), poly-γ-glutamic acid

## Abstract

In this work, we report the design and fabrication of a dual-function integrated system to monitor, in real time, the release of previously loaded 2-methyl-1,4-naphthoquinone (MeNQ), also named vitamin K3. The newly developed system consists of poly(3,4-ethylenedioxythiophene) (PEDOT) nanoparticles, which were embedded into a poly-γ-glutamic acid (γ-PGA) biohydrogel during the gelling reaction between the biopolymer chains and the cross-linker, cystamine. After this, agglomerates of PEDOT nanoparticles homogeneously dispersed inside the biohydrogel were used as polymerization nuclei for the in situ anodic synthesis of poly(hydroxymethyl-3,4-ethylenedioxythiophene) in aqueous solution. After characterization of the resulting flexible electrode composites, their ability to load and release MeNQ was proven and monitored. Specifically, loaded MeNQ molecules, which organized in shells around PEDOT nanoparticles agglomerates when the drug was simply added to the initial gelling solution, were progressively released to a physiological medium. The latter process was successfully monitored using an electrode composite through differential pulse voltammetry. The fabrication of electroactive flexible biohydrogels for real-time release monitoring opens new opportunities for theranostic therapeutic approaches.

## 1. Introduction

Over the last decade, hydrophilic polymers organized in self-assembled and/or cross-linked hydrogels were widely studied considering a great variety of physical formats, including microparticles [1,2], nanoparticles [3,4], and films [5,6,7]. In contrast to self-assembled hydrogels, which are typically made of low-molecular-weight compounds interacting through non-covalent interactions (i.e., hydrogen bonding, van der Waals, charge transfer, dipole–dipole, π–π stacking, and coordination interactions), cross-linked hydrogels are based on covalent bonds. More specifically, in the latter structures, non-covalent interactions play a less important role, mainly related to the packing of non-cross-linked segments. Thus, physical interactions usually provide reversible gel-to-phase transitions in self-assembled hydrogels, which are sometimes undesired for biomedical applications, while cross-linked hydrogels remain stable despite environmental changes. On the other hand, as a result of the progress in chemical design and synthesis, hydrogels were used in many biomedical applications, for example, tissue engineering [8,9], regenerative medicine [6,9,10], cellular immobilization [11], biosensors [1,12,13], and drug release [3,4,7,14,15].

In recent years, hydrogels based on hydrophilic poly-γ-glutamic acid (γ-PGA) attracted our attention due to their particular properties. γ-PGA is an anionic poly(γ-peptide) linked by the peptide bond between the α-amino and the γ-carboxyl groups of d- and l-glutamic acid. This homopeptide, which is naturally synthetized as a slime layer by a variety of members of the genus *Bacillus* [16,17,18], is water-soluble, biodegradable, edible, and non-toxic toward humans and the environment. Moreover, in its free acid form, γ-PGA can be chemically cross-linked, resulting in a biohydrogel with an internal architecture that can be manipulated to retain drugs, peptides, or proteins within the cross-linked network [19,20,21].

**Scheme 1:**
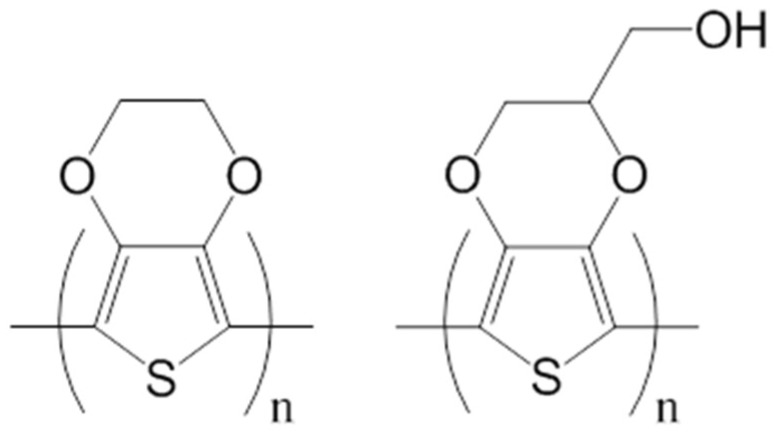
Chemical structure of poly(3,4-ethylenedioxythiohene) (PEDOT; **left**) and poly(hydroxymethyl-3,4-ethylenedioxythiophene) (PHMeEDOT; **right**).

On the other hand, conducting polymers (CPs) are also employed in a variety of biomedical applications, such as scaffolds for tissue regeneration [22,23], artificial muscles [24,25,26], drug delivery systems [27,28,29], and biosensors [30,31,32,33], among others. Among CPs, polythiophene (PTh) and its derivatives are particularly important due to their high stability, excellent electrical and electrochemical properties, and easy functionalization [34]. Recently, our research group studied the application of poly(3,4-ethylenedioxythiohene) and poly(hydroxymethyl-3,4-ethylenedioxythiophene), abbreviated PEDOT and PHMeEDOT (Scheme 1), respectively, as electroactive platforms for the detection of neurotransmitters like dopamine (DA) [31,32] and serotonin (SRT) [32], both related with different neuronal pathologies.

**Scheme 2:**
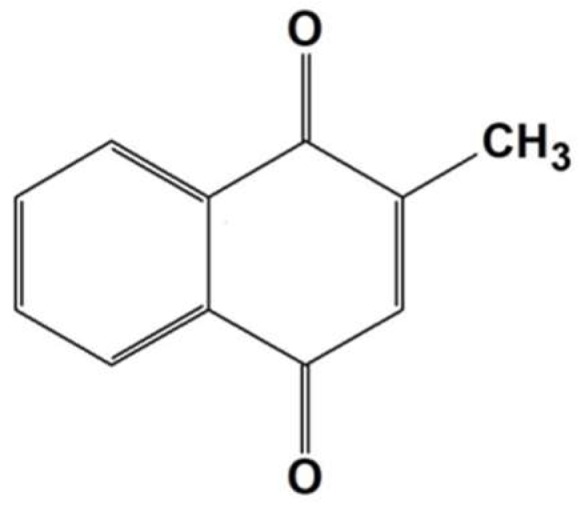
Chemical structure of 2-methyl-1,4-naphthoquinone (MeNQ).

Another important biomolecule in the human body is 2-methyl-1,4-naphthoquinone (MeNQ), also called vitamin K3 or menadione (Scheme 2). This synthetic blood coagulation vitamin with fat solubility is mainly used as a component of multivitamin drugs with anti-hemorrhagic activity [35,36]. However, it is also involved in photosynthetic mechanisms [37], cellular respiration [38], oxidative phosphorylation [39], and anticancer processes [40], due to its ability to transport electrons and protons.

In this work, we propose a flexible bioplatform for the simultaneous detection and release of MeNQ. This dual-functionalization strategy offers a very promising design principle since the integration of real-time MeNQ monitoring and release opens a new door for the development of bioplatforms for theranostic therapeutics. More specifically, the γ-PGA biohydrogel matrix was used as a flexible solid support and to load both the hydrophobic drug and PEDOT nanoparticles (PEDOT NPs), the latter being used as nuclei for the in situ anodic polymerization of PHMeEDOT. In the resulting multicomponent bioplatform, the CPs, PEDOT, and PHMeEDOT electrochemically detect the MeNQ, while, at the same time, it is released from the γ-PGA matrix to the physiologic medium.

## 2. Results and Discussion

### 2.1. Loading of PEDOT NPs into γ-PGA Hydrogels

Firstly, PEDOT NPs and MeNQ were loaded into a biocompatible γ-PGA hydrogel. For this purpose, PEDOT NPs were prepared using an aqueous micellar dispersion of 4-dodecylbenzenesulfonic acid (DBSA), 3,4-ethylenedioxythiophene (EDOT) monomer, and ammonium persulfate (APS). The complete experimental procedure, which was adapted from that of Puiggalí-Jou et al. [27], is described in Section 4. PEDOT NPs were loaded into the γ-PGA hydrogel by incorporating them (20% *w*/*w* with respect to the weight of γ-PGA) into the 0.5 M NaHCO_3_ solution used to dissolve the biopolymer and the 1-[3(dimethylamino)propyl]-3-ethylcarbodiimide methiodide (EDC), which was used to activate the carboxylic acid groups. After this, cystamine dihydrochloride was added to the solution to form the cross-links. Hereafter, the resulting PEDOT NP-loaded hydrogel is denoted as γ-PGA/PEDOT.

**Figure 1:**
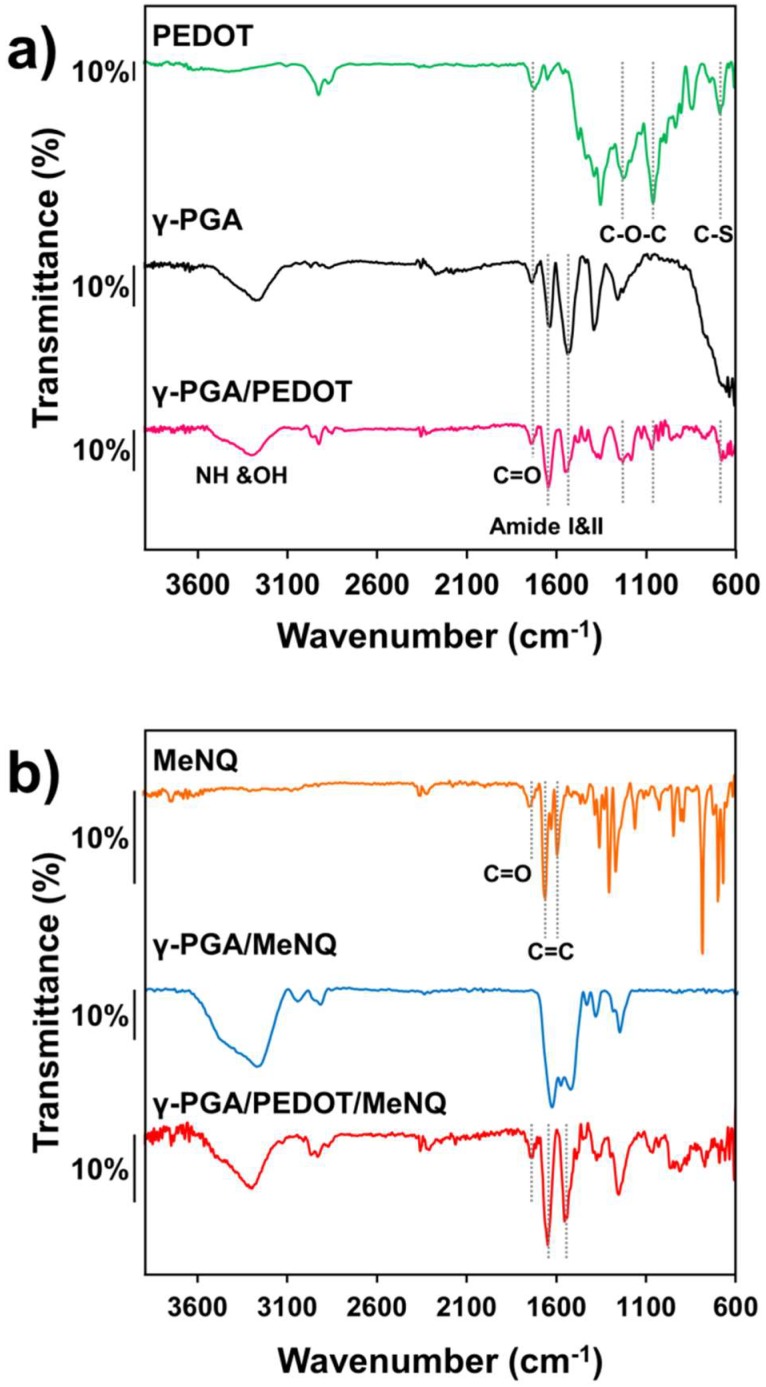
Fourier-transform infrared (FTIR) spectra of (**a**) poly(3,4-ethylenedioxythiophene) nanoparticles (PEDOT NPs), poly-γ-glutamic acid (γ-PGA), and γ-PGA/PEDOT; and of (**b**) 2-methyl-1,4-naphthoquinone (MeNQ), γ-PGA/MeNQ, and γ-PGA/PEDOT/MeNQ. Grey dotted lines indicate the position of the most relevant bands (see main text).

The Fourier-transform infrared (FTIR) spectra of PEDOT NPs, unloaded γ-PGA, and γ-PGA/PEDOT are compared in Figure 1a. The spectrum recorded for the nanoparticles shows the characteristic bands of PEDOT: thiophene ring fundamental vibrations present at 1643 and 1553 cm^−1^; C–S stretching at 681 cm^−1^; and vibrational bands associated to the C–O–C stretching from the ethylenedioxy group at 1233 and 1053 cm^−1^. The PEDOT NP spectrum also allows identifying the dodecylbenzene sulfonic acid (DBSA) dopant. Thus, the peaks located between 2863 and 2917 cm^−1^ were associated with the CH_3_, CH_2_, and CH stretch, while the absorption in the range of 1000–1400 cm^−1^ corresponds to the SO_3_^−^ groups of DBSA, overlapping the C–O–C stretching bands of PEDOT. Furthermore, the peak at 1711 cm^−1^ indicates the existence of carbonyl groups, which were attributed to the overoxidation of the polymer [27,41,42,43].

The spectrum recorded for the biopolymer shows a broad band located between 3100 and 3500 cm^−1^, which was attributed to N–H and O–H stretching vibrations, and two intense but narrow peaks at 1640 and 1539 cm^−1^, which correspond to the stretching vibration of the amide carbonyl group (amide I) and the CO–NH bond vibration (amide II) at 1539 cm^−1^. The small band associated with the free carboxylic acid at 1718 cm^−1^ and the lack of asymmetric COO^−^ bands at 1595 cm^−1^ reflect the successful formation of CO–NH bonds due to the reaction between the biopolymer and the cross-linker [19,44]. As expected, the spectrum of the γ-PGA/PEDOT composite contains the principal bands associated with γ-PGA, as well as those related to the DBSA dopant and PEDOT NPs.

On the other hand, the loading of MeNQ was performed by adding the drug (10 mM) to the solution used for the preparation of γ-PGA and γ-PGA/PEDOT hydrogels, with the resulting composites being denoted as γ-PGA/MeNQ and γ-PGA/PEDOT/MeNQ, respectively. Figure 1b compares the FTIR spectra of MeNQ, γ-PGA/MeNQ, and γ-PGA/PEDOT/MeNQ. The MeNQ powder reflects principal bands at 1732, 1586, and 1653 cm^−1^, which were attributed to the C=O stretching vibration, the quinone C=C stretching vibration, and C=C stretching of the aromatic ring, respectively [45]. Unfortunately, these peaks are barely appreciated in the γ-PGA/MeNQ and γ-PGA/PEDOT/MeNQ spectra due of the overlap with the biopolymer bands.

**Figure 2:**
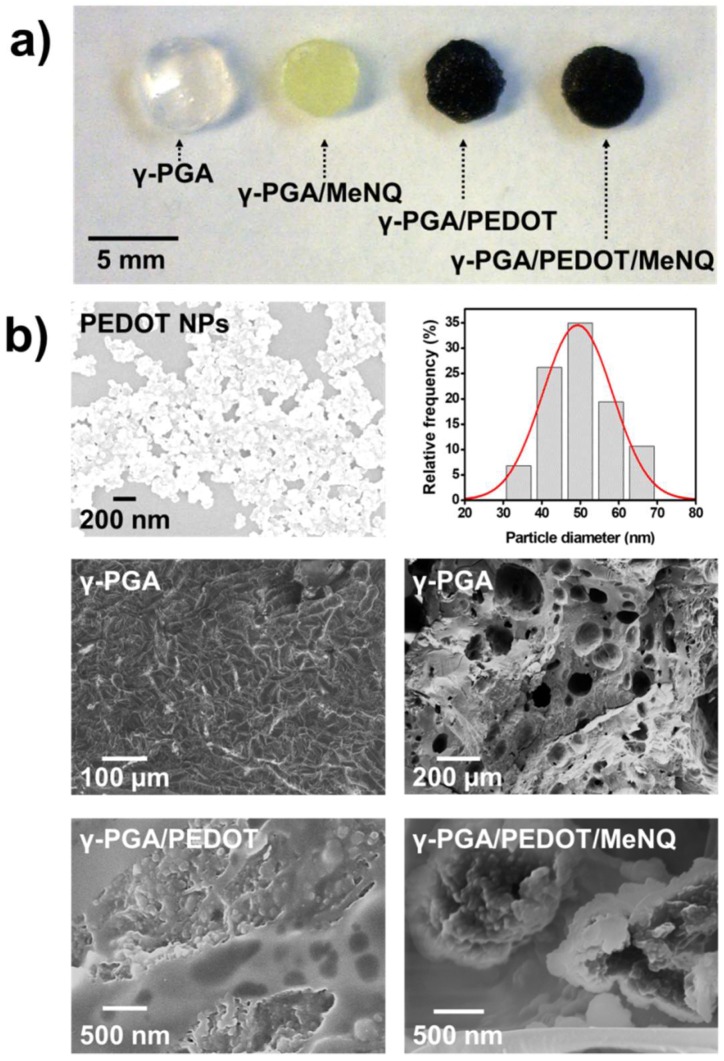
(**a**,**b**) SEM micrographs of PEDOT NPs, γ-PGA (right, cross-section), γ-PGA/PEDOT, and γ-PGA/PEDOT/MeNQ. The size distribution histogram of PEDOT NPs as determined from SEM measurements is also displayed (i.e., around 200 measures from different representative SEM micrographs were performed).

The appearances of γ-PGA, γ-PGA/PEDOT, γ-PGA/MeNQ, and γ-PGA/PEDOT/MeNQ are displayed in Figure 2a. The incorporation of PEDOT NPs and MeNQ resulted in a homogeneous color change. Thus, γ-PGA adopts a yellow coloration upon the incorporation of MeNQ, while the hydrogel matrix turns into dark blue when the PEDOT NPs are incorporated. Representative micrographs of the different samples studied in this work are displayed in Figure 2b. As can be seen, PEDOT NPs show a coral-like morphology with an effective diameter of 49 ± 7 nm. On the other hand, loaded hydrogels retain the typical porous structure of unloaded γ-PGA, which shows pore sizes of between ~23 and 200 μm. Although similar structures were observed for γ-PGA/PEDOT and γ-PGA/PEDOT/MeNQ, higher-magnification micrographs show that the PEDOT NPs form agglomerates homogeneously dispersed into the hydrogel matrix. Moreover, the presence of MeNQ creating a shell around PEDOT NPs agglomerates was detected in γ-PGA/PEDOT/MeNQ. The contact between the CP and the drug through such a shell is expected to facilitate the release of MeNQ upon electrostimulation. Thus, the applied potential should modulate the strength of the PEDOT···MeNQ interactions by altering the electronic structure of the CP. It is worth noting that the good distribution of PEDOT NPs, as reflected by the effective diameter histograms derived from SEM measurements (Figure 2b), presumably provides an initial point of polymerization for the chronoamperometric synthesis of PHMeEDOT in aqueous solution.

### 2.2. Characterization of Electroactive and Flexible Electrodes Prepared through PHMeEDOT Polymerization

To improve the electrochemical behavior of the flexible electrodes, hydroxymethyl-3,4-ethylenedioxythiophene (HMeEDOT) monomers in aqueous solution were anodically polymerized inside the γ-PGA/PEDOT and γ-PGA/PEDOT/MeNQ hydrogels, with the resulting composites being denoted as [γ-PGA/PEDOT]PHMeEDOT and [γ-PGA/PEDOT/MeNQ]PHMeEDOT, respectively. It should be emphasized that (i) before the polymerization, the hydrogels were kept in the polymerization medium overnight to guarantee the diffusion of the HMeEDOT monomers into the hydrogel; and (ii) HMeEDOT monomer was chosen because of its high solubility in water, which was attributed to the exocyclic hydroxymethyl group [31,44].

**Figure 3:**
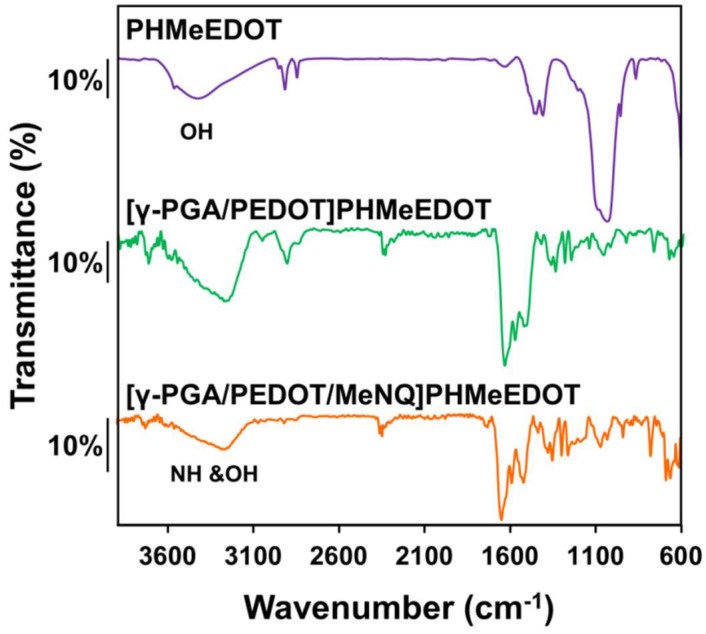
FTIR spectra of PHMeEDOT, [γ-PGA/PEDOT]PHMeEDOT, and [γ-PGA/PEDOT/MeNQ]PHMeEDOT. PHMeEDOT—poly(hydroxymethyl-3,4-ethylenedioxythiophene); γ-PGA—poly-γ-glutamic acid; PEDOT—poly(3,4-ethylenedioxythiohene); MeNQ—2-methyl-1,4-naphthoquinone.

The FTIR spectra of PHMeEDOT, [γ-PGA/PEDOT]PHMeEDOT, and [γ-PGA/PEDOT/MeNQ]PHMeEDOT are displayed in Figure 3. In addition to the bands observed for PEDOT NPs, PHMeEDOT presents a broad band at 3428 cm^−1^, which was associated to the exocyclic OH group. On the other hand, all electrodes display the γ-PGA hydrogel bands (Figure 1). Unfortunately, the OH band from PHMeEDOT is not clearly identified in the [γ-PGA/PEDOT]PHMeEDOT and [γ-PGA/PEDOT/MeNQ]PHMeEDOT spectra since it overlaps with the backbone NH and the side carboxylic OH bands from γ-PGA.

**Figure 4:**
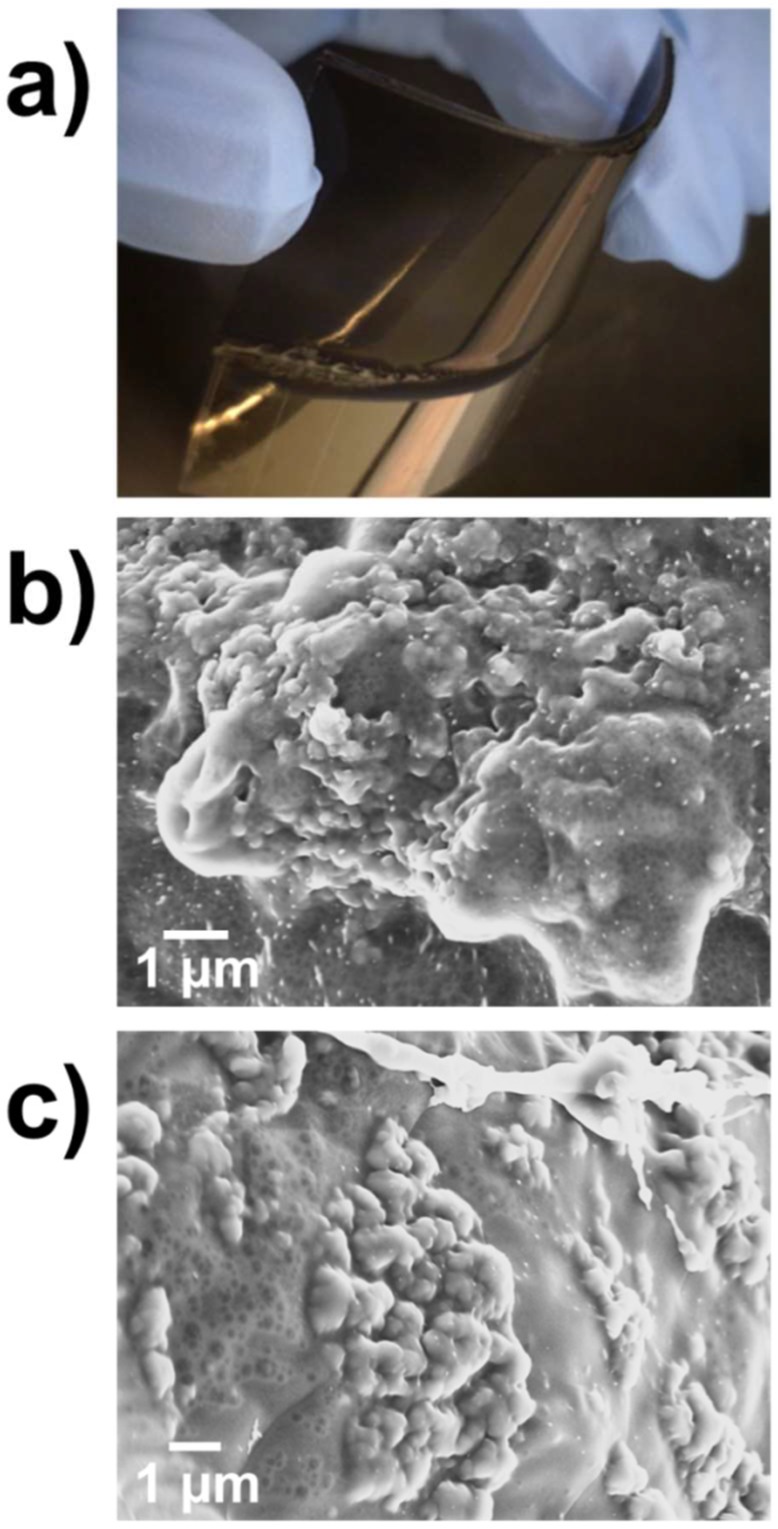
(**a**) Photograph of the flexible [γ-PGA/PEDOT/MeNQ]PHMeEDOT electrode. SEM micrographs of (**b**) [γ-PGA/PEDOT]PHMeEDOT, and (**c**) [γ-PGA/PEDOT/MeNQ]PHMeEDOT. γ-PGA—poly-γ-glutamic acid; PEDOT—poly(3,4-ethylenedioxythiohene); PHMeEDOT—poly(hydroxymethyl-3,4-ethylenedioxythiophene); MeNQ—2-methyl-1,4-naphthoquinone.

Figure 4a shows the physical appearance of flexible [γ-PGA/PEDOT/MeNQ]PHMeEDOT electrodes, which were attached to indium tin oxide (ITO)-coated polyethylene terephthalate (PET; sheet resistance: 75 Ω) for the polymerization of HMeEDOT. Although, the incorporation of PHMeEDOT into the hydrogels did not cause any apparent visual change, the weight increased by ~9% and ~15% for [γ-PGA/PEDOT]PHMeEDOT and [γ-PGA/PEDOT/MeNQ]PHMeEDOT, respectively. This increment is consistent with the growth of PHMeEDOT chains inside the hydrogels. Furthermore, high-magnification SEM micrographs indicate that the morphology of the electroactive hydrogels was also affected by the electropolymerization (Figure 4b,c). In both cases, the NP agglomerates acted as polymerization nuclei, which were covered by PHMeEDOT chains forming a thin layer inside the biocompatible matrix. Moreover, the swelling ratio (SR) of the different studied systems, which is shown in Table 1, changed upon the incorporation of PHMeEDOT. More specifically, the ability to absorb water, which was already affected by the incorporation of PEDOT NPs, increased noticeably after the anodic polymerization process. These effects were attributed to the hydrophilicity of PEDOT and, specially, of PHMeEDOT. Additionally, this effect was smaller in MeNQ-loaded systems due to the hydrophobicity of this drug.

**Figure 5:**
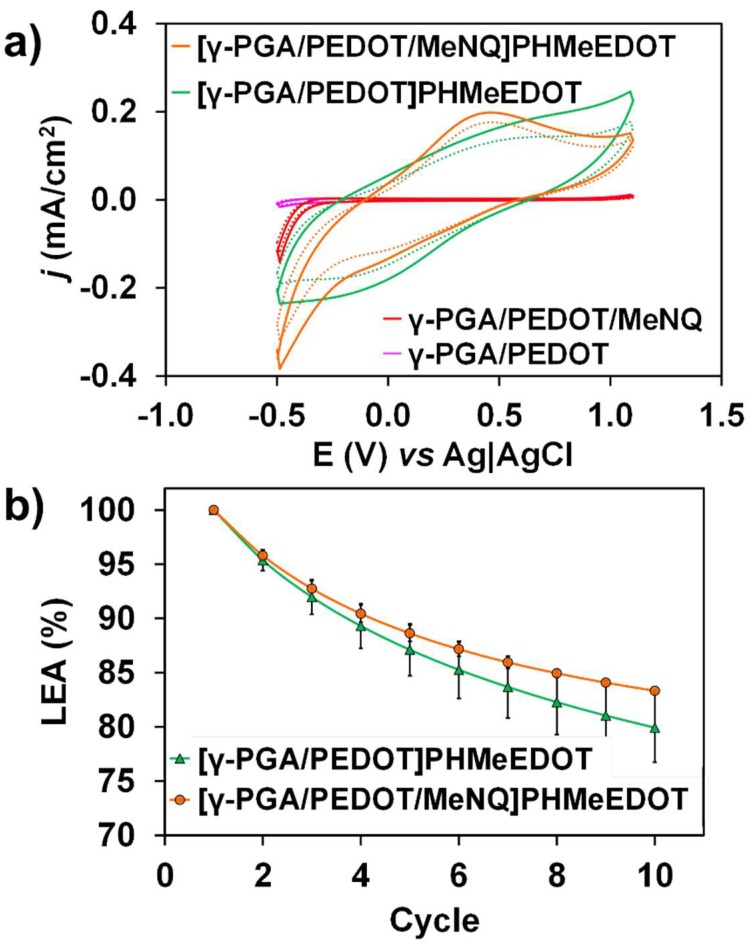
(**a**) First control voltammogram (solid lines) and voltammogram after 10 consecutive oxidation–reduction cycles (dashed lines) in 0.1 M phosphate-buffered saline (PBS) for γ-PGA/PEDOT, γ-PGA/PEDOT/MeNQ, [γ-PGA/PEDOT]PHMeEDOT, and [γ-PGA/PEDOT/MeNQ]PHMeEDOT. (**b**) Loss of electrochemical activity (LEA) against 10 consecutive redox cycles for [γ-PGA/PEDOT]PHMeEDOT and [γ-PGA/PEDOT/MeNQ]PHMeEDOT. γ-PGA—poly-γ-glutamic acid; PEDOT—poly(3,4-ethylenedioxythiohene); PHMeEDOT—poly(hydroxymethyl-3,4-ethylenedioxythiophene); MeNQ—2-methyl-1,4-naphthoquinone.

The electroactivity and electrochemical stability of the prepared electrodes were determined by cyclic voltammetry (CV) in 0.1 M phosphate-buffered saline (PBS) solution (pH = 7.4). The voltammogram recorded for the γ-PGA hydrogel (not shown) was similar in both shape and area to the one obtained for γ-PGA/PEDOT (Figure 5a), indicating that the incorporation of PEDOT NPs into the dielectric hydrogel matrix did not cause an increment in the electrochemical activity. The γ-PGA/PEDOT/MeNQ behavior was also similar.

On the other hand, the control voltammogram of [γ-PGA/PEDOT]PHMeEDOT resembles that already reported for neat PHMeEDOT [31]. The anodic peak at around +0.7 V and a cathodic peak at −0.02 V were interpreted as the reversible formation of polarons in the CP polymer chains. Furthermore, the [γ-PGA/PEDOT/MeNQ]PHMeEDOT voltammogram displays an anodic peak at +0.35 V associated with the oxidation of the immobilized MeNQ.

It is worth noting that the anodic polymerization of HMeEDOT into the hydrogel matrix causes a remarkable improvement of the electrochemical properties in the hydrogel composites. Thus, the cathodic and anodic areas of the voltammograms recorded for [γ-PGA/PEDOT]PHMeEDOT and [γ-PGA/PEDOT/MeNQ]PHMeEDOT are significantly greater than those for γ-PGA/PEDOT and γ-PGA/PEDOT/MeNQ. Moreover, the charge stored in the two PHMeEDOT-containing systems are very similar (Figure 5b), indicating that the electrochemical improvement is not due to the presence of MeNQ, but to the CP. After 10 consecutive oxidation–reduction cycles, the losses of electroactivity for [γ-PGA/PEDOT]PHMeEDOT and [γ-PGA/PEDOT/MeNQ]PHMeEDOT were 20% and 17%, respectively.

### 2.3. Release and Detection of MeNQ

**Figure 6:**
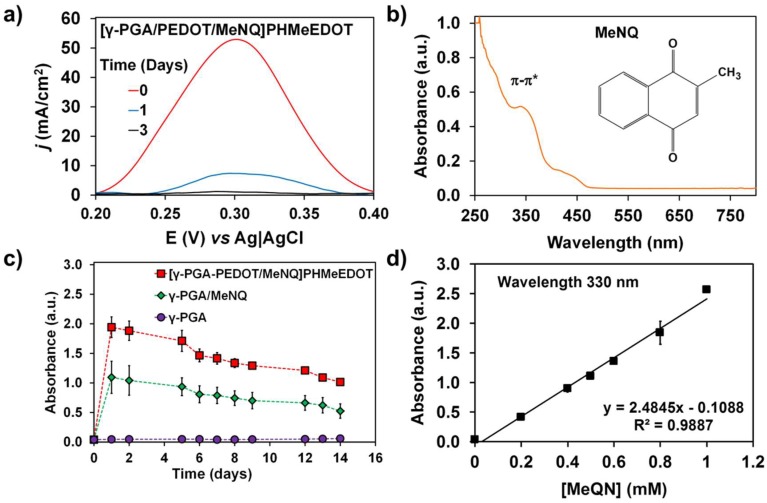
(**a**) Differential pulse voltammograms recorded for [γ-PGA/PEDOT/MeNQ]PHMeEDOT after immersion for 0, 1, and 3 days in a 0.1 M PBS solution. (**b**) Ultraviolet–visible light (UV–Vis) spectrum and chemical structure (inset) of 2-methyl-1,4-naphthoquinone (MeNQ). (**c**) Kinetics of the MeNQ release from γ-PGA, γ-PGA/MeNQ, and [γ-PGA/PEDOT/MeNQ] PHMeEDOT, as determined by UV–Vis spectroscopy. (**d**) Calibration curve for MeNQ in a concentration range of 0 to 1 mM. γ-PGA—poly-γ-glutamic acid; PEDOT—poly(3,4-ethylenedioxythiohene); PHMeEDOT—poly(hydroxymethyl-3,4-ethylenedioxythiophene).

The electroactivity of [γ-PGA/PEDOT/MeNQ]PHMeEDOT is suitable for determining the evolution of the loaded MeNQ when the hydrogel is immersed in a 0.1 M PBS solution at pH = 7.4. Voltammograms recorded by differential pulse voltammetry (DPV) before and after 1 and 3 days of immersion are displayed in Figure 6a. Although the peak associated with the oxidation of MeNQ was observed at +0.35 V in all cases, the peak current density decreased considerably with time. The reduction in the amount of drug inside the hydrogel demonstrates not only the release capacity of the [γ-PGA/PEDOT/MeNQ]PHMeEDOT electrode, but also its ability to detect such a release.

On the other hand, the release of MeNQ from γ-PGA/MeNQ and [γ-PGA/PEDOT/ MeNQ]PHMeEDOT was evaluated by ultraviolet–visible light (UV–Vis) spectroscopy at a wavelength of 330 nm. It is worth nothing that such a wavelength corresponds to the π–π* transition of MeNQ [46,47], as reflected in the UV–Vis spectrum displayed in Figure 6b. For these assays, MeNQ-loaded hydrogels were immersed in 0.1 M PBS (pH = 7.4) solutions, and aliquots from such solutions were measured at different times. Figure 6c compares the behavior of γ-PGA, γ-PGA/MeNQ, and [γ-PGA/PEDOT/MeNQ]PHMeEDOT along 14 days of immersion in physiological medium. As expected, measurements for γ-PGA resulted in a constant absorbance, indicating no compound being released from the biopolymer that could interfere with the experiment. In contrast, γ-PGA/MeNQ and [γ-PGA/PEDOT/MeNQ]PHMeEDOT exhibited an enlargement in the absorbance lectures after one day (i.e., from 0 to ~1.1 and ~1.9, respectively), reflecting the increase in concentration of MeNQ in the medium. After that, the absorbance decreased slowly with time. Two different explanations can be considered for this behavior: (i) the release of MeNQ from the hydrogel toward the solution competes with the opposite process, which consists of the re-loading of the drug from the solution into the hydrogel; or (ii) the MeNQ oxidizes with time, and the absorption peak associated with π–π* transition shifts a little bit with respect to the wavelength of 330 nm used for measurements. The standard calibration (STC) curve displayed in Figure 6d allowed us to determine that the concentration of MeNQ released was 0.4 mM for γ-PGA/MeNQ and 0.9 mM for [γ-PGA/PEDOT/MeNQ]PHMeEDOT after 24 h.

Overall, the results displayed in Figure 6 corroborate that the combination of CPs and biopolymers is very useful for the development of advanced biomedical devices, for example, systems able to release drugs to the medium under real-time monitoring by electrochemical detection. In this work, we combined the properties of γ-PGA hydrogels with the electroactivity of both PEDOT and PHMeEDOT to synthesize a flexible electrode capable of following the release of previously loaded MeNQ.

## 3. Conclusions

An MeNQ release system able to monitor the concentration of the delivered drug was developed by incorporating CPs into a γ-PGA biohydrogel. The characterization of this integrated system proves not only the MeNQ-loading capability of the γ-PGA biohydrogel, but also the detection capacity of the integrated CPs, which consists of PEDOT NPs coated with anodically polymerized PHMeEDOT chains. This integrated system is very promising for the development of theranostic therapeutic systems for a wide variety of diseases in which MeNQ is used as the active principle. Although much work is still required to use this integrated system as a tool to strictly control the MeNQ release, the reported strategy offers an excellent opportunity for the development of more advanced and sophisticated devices.

## 4. Materials and Methods

### 4.1. Materials

DBSA, APS, EDOT, HMeEDOT, cystamine dihydrochloride (≥98.0%), 1-[3(dimethylamino)propyl]-3-ethylcarbodiimide methiodide (EDC), 2-methyl-1,4-naphthoquinone (MeNQ), and anhydrous lithium perchlorate (LiClO_4_) were purchased from Sigma-Aldrich Chemical Company. LiClO_4_ was dried in an oven at 70 °C before use in electrochemical experiments. Sodium bicarbonate (NaHCO_3_) was purchased from Panreac Quimica S.A.U. (Barcelona, Spain). Free-acid γ-PGA (from *Bacillus subtilis*), with an average molecular weight of M_w_ = 350,000, was purchased from Wako Chemicals GmbH (Neuss, Germany).

### 4.2. Synthesis of PEDOT NPs

PEDOT NPs were prepared by adapting the procedure reported by Puiggalí-Jou et al. [27]. Aqueous micellar dispersion was prepared by stirring (750 rpm) 0.07 g of DBSA in 20 mL of milli-Q water for 1 h. Then, 11.8 mg of EDOT monomer was added to the DBSA micellar solution and solubilized with stirring. After 1 h, 0.45 g of the oxidizer APS was dissolved in 5 mL of milli-Q water and, then, added to the solution. The reaction temperature was kept at 30 °C and stirred for 24 h. Along this time, the reaction mixture turned from a light gray color to dark blue.

After polymerization, no sedimentation was observed, indicating good colloidal stability. The resulting solution was centrifuged at 11,000 rpm for 40 min at 4 °C. Then, the supernatant solution was decanted, and the sediment was re-dispersed in milli-Q water using an ultrasonic bath for 15 min at 30 °C. In order to ensure the removal of unreacted chemicals and to purify the dispersion medium, the centrifugation and re-dispersion processes were performed two more times each. Finally, the pellet obtained was left under vacuum for two days.

### 4.3. Synthesis of the γ-PGA Hydrogel

γ-PGA biohydrogels were synthesized adapting procedures previously reported [19,20]. γ-PGA and EDC were dissolved in 1 mL of 0.5 M NaHCO_3_ at 4 °C and 500 rpm for 12 min. Then, cystamine dihydrochloride was added to the solution and mixed in the same conditions for 2 min. The γ-PGA/EDC/cystamine molar ratio was 5/4/2. The final solution was removed with a magnetic stirrer and relocated into glass molds of 4 × 1 × 0.1 cm^3^, and was left to gel at room temperature for 30 min. To remove any compound in excess, the resulting hydrogel was washed three times with a 0.1 M PBS solution (pH = 7.4).

### 4.4. Preparation of γ-PGA/PEDOT

PEDOT-containing γ-PGA hydrogels were prepared following the procedure described for γ-PGA hydrogels, but after introducing the following changes: (i) 20% *w*/*w* PEDOT NPs with respect to the weight of γ-PGA was incorporated into the 0.5 M NaHCO_3_ solution; and (ii) the biopolymer was dissolved by stirring the PEDOT NP-containing solution for 12 h at 1000 rpm.

### 4.5. Preparation of [γ-PGA/PEDOT]PHMeEDOT Composites

A sheet of ITO-coated PET (4 × 3 cm^2^) was coated with the γ-PGA/PEDOT hydrogel previously prepared. The flexible working electrode was kept for 12 h in an aqueous solution of 10 mM HMeEDOT and 0.1 M LiClO_4_. After this, an anodic polymerization was carried out in a three-electrode cell at a constant potential of +1.10 V and adjusting the polymerization charge to 20 mC. It is worth noting that, although DBSA was utilized as a dopant agent for the chemical polymerization of PEDOT, LiClO_4_ is a more appropriate dopant agent for the electrochemical polymerization of PHMeDOT because of its greater mobility. The polymerization was conducted on a potentiostat-galvanostat Autolab PGSTAT302N using Ag|AgCl 3 M KCl as a reference electrode and a steel AISI 316 sheet (1 cm^2^) as a counter electrode.

### 4.6. Preparation of MeNQ-Containing Hydrogels

MeNQ was incorporated into γ-PGA, γ-PGA/PEDOT, and [γ-PGA/PEDOT]PHMeEDOT hydrogels. For this purpose, MeNQ was added to a 0.5 M NaHCO_3_ solution (10 mM) and mixed at 1000 rpm for 24 h. This solution was used for the preparation of the different hydrogels using the procedure previously described.

### 4.7. Characterization

FTIR spectra were recorded on a FTIR Jasco 4100 spectrophotometer. The samples were deposited on an attenuated total reflection accessory (top-plate) with a diamond crystal (Specac model MKII Golden Gate Heated Single Reflection Diamond ATR). Samples were evaluated using the spectra manager software and, for each sample, 32 scans were performed between 4000 and 600 cm^−1^ with a resolution of 4 cm^−1^.

Scanning electron microscopy (SEM) was used to study the surface morphology. Micrographs were obtained using a focused ion beam Zeiss Neon 40 scanning electron microscope operating at 5 kV, equipped with an energy dispersive X-ray (EDX) spectroscopy system.

The swelling ratio (*SR*, in %) of the hydrogels was determined according to

(1) $$SR=\frac{W_{w}-W_{d}}{W_{d}}\;\times \;100,$$

where *W_w_* is the weight of the hydrogels after 30 min in milli-Q water, and *W_d_* is the weight of the lyophilized hydrogel. All experiments were conducted at room temperature.

Cyclic voltammetry (CV) assays for electrochemical characterization were performed using the Autolab PGSTAT302N. Experiments were conducted in a 0.1 M PBS solution (pH = 7.4) at room temperature. The initial and final potentials were −0.5 V, while the reversal potential was +1.1 V. A scan rate of 100 mV·s^−1^ was applied in all cases. Ag|AgCl 3 M KCl and steel AISI 316 sheets of 1 cm^2^ were used as reference and counter electrodes, respectively. The different working electrodes were obtained as follows: (1) the hydrogels under study were prepared as described above in a glass mold with dimensions of 4 (length) × 3 (width) × 0.1 (height) cm^3^; (2) the hydrogels were removed from the mold and washed three times (20 min each time) in 0.1 M PBS (pH 7.4); and (3) the washed hydrogels were directly attached to flexible ITO-coated PET sheets (4 × 3 cm^2^).

The ability to exchange charge reversibly (i.e., electroactivity) and the electrochemical stability (i.e., electrostability) were determined through direct measurement of the anodic and cathodic areas in the control voltammograms using NOVA software (Metrohm Autolab B.V., Utrech, The Netherlands). The loss of electroactivity (LEA, in %) was expressed as

(2) $$LEA=\frac{\Delta Q}{Q_{II}}\;\times \;100,$$

where Δ*Q* is the difference in voltammetric charges (in C) between the second and the last cycle, and *Q_II_* is the voltammetric charge corresponding to the second cycle.

### 4.8. Release and Electrochemical Detection of MeNQ

DPV was applied for the electrochemical detection of MeNQ. Assays were carried out in a three-electrode cell, as that described for CV assays, with fresh PBS solution, a potential range between +0.2 V and +0.4 V, 80 mV as modulation amplitude, and a scan increment of 2 mV. The modulation and interval time were 0.05 s and 40 s, respectively. The DPV signals corresponding to electrochemical oxidation of MeNQ in the hydrogels were obtained after 0, 1, and 3 days in PBS solution (pH 7.4) at 37 °C and 80 rpm.

Spectroscopic studies regarding the MeNQ release were performed considering three different samples (0.5 × 2 × 0.1 cm^3^) of the following hydrogels: γ-PGA, γ-PGA/MeNQ and [γ-PGA/PEDOT/MeNQ] PHMeEDOT. Samples were immersed in a 0.1 M PBS solution (pH = 7.4) for 14 days at 37 °C and 80 rpm. Periodically, UV–Vis spectra were recorded in a UV–Vis Cary 100 Bio spectrophotometer (Agilent, Santa Clara, CA, USA).

## Figures and Tables

**Table 1:** Swelling ratio (SR; %) of the different hydrogels determined using Equation (1). γ-PGA—poly-γ-glutamic acid; PEDOT—poly(3,4-ethylenedioxythiohene); PHMeEDOT—poly(hydroxymethyl-3,4-ethylenedioxythiophene); MeNQ—2-methyl-1,4-naphthoquinone.

Sample	SR (%)
*γ*-PGA	408 ± 170
γ-PGA/PEDOT	488 ± 254
[γ-PGA/PEDOT]PHMeEDOT	922 ± 92
γ-PGA/MeNQ	624 ± 81
γ-PGA/PEDOT/MeNQ	932 ± 375
[γ-PGA/PEDOTMeNQ]PHMeEDOT	1711 ± 82

## References

[1] Le Goff G.C., Srinivas R.L., Hill W.A., Doyle P.S. (2015). Hydrogel microparticles for biosensing. Eur. Polym. J..

[2] Choi D., Jang E., Park J., Koh W.G. (2008). Development of microfluidic devices incorporating non-spherical hydrogel microparticles for protein-based bioassay. Microfluid Nanofluidics.

[3] Kim K.H., Kim J., Jo W.H. (2005). Preparation of hydrogel nanoparticles by atom transfer radical polymerization of N-isopropylacrylamide in aqueous media using PEG macro-initiator. Polymer.

[4] Na K., Park K.-H., Kim S.W., Bae Y.H. (2000). Self-assembled hydrogel nanoparticles from curlan derivatives: Characterization, anti-cancer drug delivery and interaction with a hepatoma cell line (Hep G2). J. Control. Release.

[5] Ye Y., Mao Y. (2011). Vapor-based synthesis of ultrathin hydrogel coatings for thermo-responsive nanovalves. J. Mater. Chem..

[6] Wathoni N., Motoyama K., Higashi T., Okajima M., Kaneko T., Arima H. (2016). Physically crosslinked-sacran hydrogel films for wound dressing application. Int. J. Biol. Macromol..

[7] Zhu W., Xiong L., Wang H., Zha G., Du H., Li X., Shen Z. (2015). Sustained drug release from an ultrathin hydrogel film. Polym. Chem..

[8] Yu L., Ding J. (2008). Injectable hydrogels as unique biomedical materials. Chem. Soc. Rev..

[9] Peppas N.A., Hilt J.Z., Khademhosseini A., Langer R. (2006). Hydrogels in biology and medicine: From molecular principles to bionanotechnology. Adv. Mater..

[10] Annabi N., Tamayol A., Uquillas J.A., Akbari M., Bertassoni L.E., Cha C., Camci-Unal G., Dokmeci M.R., Peppas N.A., Khademhosseini A. (2014). 25th anniversary article: Rational design and applications of hydrogels in regenerative medicine. Adv. Mater..

[11] Tan W.H., Takeuchi S. (2007). Monodisperse alginate hydrogel microbeads for cell encapsulation. Adv. Mater..

[12] Jung I.Y., Kim J.S., Choi B.R., Lee K., Lee H. (2017). Hydrogel Based Biosensors for In Vitro Diagnostics of Biochemicals, Proteins, and Genes. Adv. Healthc. Mater..

[13] Tavakoli J., Tang Y. (2017). Hydrogel based sensors for biomedical applications: An updated review. Polymers.

[14] Hoare T.R., Kohane D.S. (2008). Hydrogels in drug delivery: Progress and challenges. Polymer.

[15] Yang Z., Zhang Y., Markland P., Yang V.C. (2002). Poly(glutamic acid) poly(ethylene glycol) hydrogels prepared by photoinduced polymerization: Synthesis, characterization, and preliminary release studies of protein drugs. J. Biomed. Mater. Res..

[16] Thorne C.B., Gomez C.G., Noyes H.E., Housewright R.D. (1954). Production of glutamyl polypeptide by Bacillus subtilis. J. Bacteriol..

[17] Shih I.L., Van Y.T. (2001). The production of poly-(gamma-glutamic acid) from microorganisms and its various applications. Bioresour. Technol..

[18] Bajaj I., Singhal R. (2011). Poly (glutamic acid)—An emerging biopolymer of commercial interest. Bioresour. Technol..

[19] Pérez-Madrigal M.M., Edo M.G., Díaz A., Puiggalí J., Alemán C. (2017). Poly-γ-glutamic Acid Hydrogels as Electrolyte for Poly(3,4-ethylenedioxythiophene)-Based Supercapacitors. J. Phys. Chem. C.

[20] Matsusaki M., Serizawa T., Kishida A., Akashi M. (2005). Novel functional biodegradable polymer. III. The construction of poly(γ-glutamic acid)-sulfonate hydrogel with fibroblast growth factor-2 activity. J. Biomed. Mater. Res. Part A.

[21] Fabregat G., Giménez A., Díaz A., Puiggalí A., Alemán C. (2018). Dual-functionalization device for therapy through dopamine release and monitoring. Macromol. Biosci..

[22] Lee J.W. (2014). Porous-structured conductive polypyrrole cell scaffolds. Bull. Korean Chem. Soc..

[23] Zhao Q., Jamal R., Zhang L., Wang M., Abdiryim T. (2014). The structure and properties of PEDOT synthesized by template-free solution method. Nanoscale Res. Lett..

[24] Kim J., Deshpande S.D., Yun S., Li Q. (2006). A Comparative Study of Conductive Polypyrrole and Polyaniline Coatings on Electro-Active Papers. Polym. J..

[25] Moschou E.A., Peteu S.F., Bachas L.G., Madou M.J., Daunert S. (2004). Artificial muscle material with fast electroactuation under neutral pH conditions. Chem. Mater..

[26] Stoyanov H., Kollosche M., Risse S., Waché R., Kofod G. (2013). Soft conductive elastomer materials for stretchable electronics and voltage controlled artificial muscles. Adv. Mater..

[27] Puiggalí-Jou A., Micheletti P., Estrany F., del Valle L.J., Alemán C. (2017). Electrostimulated Release of Neutral Drugs from Polythiophene Nanoparticles: Smart Regulation of Drug–Polymer Interactions. Adv. Healthc. Mater..

[28] George P.M., Lavan D.A., Burdick J.A., Chen C.Y., Liang E., Langer R. (2006). Electrically controlled drug delivery from biotin-doped conductive polypyrrole. Adv. Mater..

[29] Nguyen T.M., Lee S., Lee S.B. (2014). Conductive polymer nanotube patch for fast and controlled ex vivo transdermal drug delivery. Nanomedicine.

[30] Tarabella G., Balducci A.G., Coppedè N., Marasso S., D’Angelo P., Barbieri S., Cocuzza M., Colombo P., Sonvico F., Mosca R., Iannotta S. (2013). Liposome sensing and monitoring by organic electrochemical transistors integrated in microfluidics. Biochim. Biophys. Acta Gen. Subj..

[31] Fabregat G., Casanovas J., Redondo E., Armelin E., Alemán C. (2014). A rational design for the selective detection of dopamine using conducting polymers. Phys. Chem. Chem. Phys..

[32] Molina B.G., Bendrea A.D., Cianga L., Armelin E., Del Valle L.J., Cianga I., Alemán C. (2017). The biocompatible polythiophene-g-polycaprolactone copolymer as an efficient dopamine sensor platform. Polym. Chem..

[33] Molina B.G., Cianga L., Bendrea A.D., Cianga I., Del Valle L.J., Estrany F., Alemán C., Armelin E. (2018). Amphiphilic polypyrrole-poly(Schiff base) copolymers with poly(ethylene glycol) side chains: Synthesis, properties and applications. Polym. Chem..

[34] Bendrea A.D., Fabregat G., Cianga L., Estrany F., Del Valle L.J., Cianga I., Alemán C. (2013). Hybrid materials consisting of an all-conjugated polythiophene backbone and grafted hydrophilic poly(ethylene glycol) chains. Polym. Chem..

[35] Acharya B.R., Choudhury D., Das A., Chakrabarti G. (2009). Vitamin K3 disrupts the microtubule networks by binding to tubulin: A novel mechanism of its antiproliferative activity. Biochemistry.

[36] Bauer H., Fritz-Wolf K., Winzer A., Kühner S., Little S., Yardley V., Vezin H., Palfey B., Schirmer R., Davioud-Charvet E. (2006). A fluoro analogue of the menadione derivative 6-[2′-(3′-methyl)-1′,4′-naphthoquinolyl]hexanoic acid is a suicide substrate of glutathione reductase. crystal structure of the alkylated human enzyme. J. Am. Chem. Soc..

[37] Sirisha V.L., Sinha M., S’Souza J.S. (2014). Menadione-induced caspase-dependent programmed cell death in the green chlorophyte Chlamydomonas reinhardtii. J. Physiol..

[38] Zampol M.A., Barros M.H. (2018). Melatonin improves survival and respiratory activity of yeast cells challenged by alpha-synuclein and menadione. Yeast.

[39] Lim J.A., Choi S.J., Moon J.Y., Kim H.S. (2016). alpha-Syntrophin is involved in the survival signaling pathway in myoblasts under menadione-induced oxidative stress. Exp. Cell Res..

[40] Prasad C.V., Nayak V.L., Ramakrishna S., Mallavadhani U.V. (2018). Novel menadione hybrids: Synthesis, anticancer activity, and cell-based studies. Chem. Biol. Drug Des..

[41] Choi J.W., Han M.G., Kim S.Y., Oh S.G., Im S.S. (2004). Poly(3,4-ethylenedioxythiophene) nanoparticles prepared in aqueous DBSA solutions. Synth. Met..

[42] Ha Y.H., Nikolov N., Pollack S.K., Mastrangelo J., Martin B.D., Shashidhar R. (2004). Towards a transparent, highly conductive poly (3,4-ethylenedioxythiophene). Adv. Funct. Mater..

[43] Krukiewicz K., Cichy M., Ruszkowski P., Turczyn R., Jarosz T., Zak J.K., Lapkowski M., Bednarczyk-Cwynar B. (2017). Betulin-loaded PEDOT films for regional chemotherapy. Mater. Sci. Eng. C.

[44] Saborío M.C.G., Lanzalaco S., Fabregat G., Puiggalí J., Estrany F., Alemán C. (2018). Flexible Electrodes for Supercapacitors Based on the Supramolecular Assembly of Biohydrogel and Conducting Polymer. J. Phys. Chem. C.

[45] Mac M., Wirz J. (2002). Salt effects on the reactions of radical ion pairs formed by electron transfer quenching of triplet 2-methyl-1,4-naphthoquinone by amines. Optical flash photolysis and step-scan FTIR investigations. Photochem. Photobiol. Sci..

[46] Khan M.S., Khan Z.H. (2005). Ab initio and semiempirical study of structure and electronic spectra of hydroxy substituted naphthoquinones. Spectrochim. Acta Part A Mol. Biomol. Spectrosc..

[47] Zielenkiewicz W., Terekhova I.V., Kozbial M., Poznanski J., Kumeev R.S. (2007). Inclusion of menadione with cyclodextrins studied by calorimetry and spectroscopic methods. J. Phys. Org. Chem..

